# The effect of loneliness on depressive symptoms in the 65+ European population: a longitudinal observational study using SHARE data

**DOI:** 10.1007/s10433-025-00846-0

**Published:** 2025-03-13

**Authors:** Daniëlle Heleen Smit, Johan Rehnberg, Stefan Fors

**Affiliations:** 1 Aging Research Center, Karolinska Institutet and Stockholm University, Stockholm, Sweden; 2 Centre for epidemiology and community medicine, Region Stockholm, Stockholm, Sweden

**Keywords:** Loneliness, Depressive symptoms, Old age, Causal inference

## Abstract

Loneliness has consistently been found to be associated with an increased probability of depressive symptoms among older adults. Yet, the relationship is complex, and it remains unclear whether loneliness is a cause of depressive symptomatology. The aim of this study is to investigate the possible causal effect of loneliness on subsequent depressive symptoms among older adults (65+) in Europe. We analyzed two waves of observational data (2015–2017) from the Study of Health, Aging, and Retirement in Europe (SHARE) (n = 6808) and attempted to identify a causal effect of loneliness on depressive symptomatology by accounting for confounding by potential unmeasured factors using an endogenous treatment-effect model. The results showed a substantial positive association between loneliness in 2015 and depressive symptoms in 2017 in the sample. However, there was no support for the hypothesis that loneliness in 2015 was a cause of depressive symptoms in 2017. Additionally, there was no evidence of unmeasured factors confounding the relationship. Thus, loneliness may not be a cause of depressive symptoms among older adults in the short term. These findings suggest that there might be other reasons why lonely individuals are at an increased risk of depressive symptoms. Therefore, a shift in focus when aiming to reduce depressive symptoms among lonely older adults may be warranted.

**Supplementary Information:**

The online version contains supplementary material available at 10.1007/s10433-025-00846-0.

## Introduction

Loneliness and depression are two prevalent mental health concerns that are affecting aging populations worldwide (World Health Organization 2017). Up to one in three older adults in world regions, such as the USA, China, and Europe, are estimated to experience loneliness (World Health Organization 2021). A fifth of the adults aged 60 or over suffer from some form of mental health disorder, with depressive disorders being one of the leading causes of disease burden in old age globally (Ferrari et al. 2022; World Health Organization 2023). Additionally, both loneliness and depression have been associated with other negative health outcomes among older individuals, such as cardiovascular disease, functional decline, all-cause mortality, and higher healthcare costs (Hawkley and Cacioppo 2010; World Health Organization 2023). However, to date, there is limited evidence on the causal nature of the observed associations (Cacioppo et al. 2006; Coyle and Dugan 2012; Leigh-Hunt et al. 2017; Wister et al. 2022; Dahlberg et al. 2022; Luo 2023).

Older adults might face unique vulnerabilities to loneliness and depression due to retirement, social network decline, experiencing the loss of a partner, and overall health deterioration (Cacioppo et al. 2006; Gerst-Emerson and Jayawardhana 2015; Leigh-Hunt et al. 2017). As this part of the population is rapidly growing, understanding how loneliness influences negative health outcomes like depression is becoming increasingly important. Depression is a complex mental health disorder (Heikkinen and Kauppinen 2004). Besides the disease burden directly associated with depression, it has also been associated with an increase in overall morbidity, mortality, and decrease in quality of life (Heikkinen and Kauppinen 2004; World Health Organization 2017; Maier et al. 2021). There are many risk factors associated with depressive disorders, and co-occurrences with other conditions such as anxiety and loneliness are common (Heikkinen and Kauppinen 2004). As with loneliness, older adults might also be specifically vulnerable to depressive disorders as they are more susceptible to a range of associated risk factors, such as co-morbidities, physical and cognitive impairments, and bereavement (Heikkinen and Kauppinen 2004; Maier et al. 2021). Loneliness has been defined as a negative emotional state resulting from the discrepancy between someone's desired level of social connection and the level of experienced social connection and can be experienced because of, as well as despite, an individual’s social situation (Perlman and Peplau 1981; Dahlberg et al. 2022; Luo 2023). Research has estimated prevalences of old age loneliness ranging between 10% (Dahlberg et al. 2018) and 50% (Gerst-Emerson and Jayawardhana 2015). A European study by Yang and Victor (2011), who investigated loneliness prevalence across various European countries, found that in every geographic region the experienced loneliness in the 60+  population increased with age, with prevalences reaching up to 34% in the oldest age groups (Yang and Victor 2011).

Most previous studies on loneliness and depression in older adults have been of cross-sectional and observational nature and are therefore not able to fully control for potential unmeasured factors that may influence the relationship (Dahlberg et al. 2022). For example, a study by Powell et al. (2021) found that loneliness was strongly associated with a cluster of pain, fatigue, and depressive symptoms in older individuals. Taylor et al. (2018) investigated the correlation between isolation from extended family and friends, and depression and psychological hardship, and found no association between objective social isolation and depression or psychological hardship, but did find an association between subjective social isolation and depressive symptomatology and psychological distress.

Evidence from longitudinal studies that aim to investigate the loneliness–depression relationship is more limited and ambiguous. For instance, Heikkinen and Kauppinen (2004) investigated changes in depressive symptoms over a period of 10 years among Finnish 75 +  adults. They found that loneliness, as well as changes in other health-related factors, predicted the onset of depressive symptomatology (Heikkinen and Kauppinen 2004). Luo (2023) investigated bidirectional associations between loneliness as well as social isolation and depression among middle-aged and older adults. By examining 12 years of data over four waves, this bidirectional association was found between loneliness and depression, but not between social isolation and depression (Luo 2023). In contrast, a study by Cacioppo et al. (2010) found that loneliness predicted later depressive symptoms, but not the other way around. Building on the Cacioppo et al. (2010) study, Griffin et al. (2022) investigated 8 years of U.S. Health and Retirement Study data and found a strong association between the trait-like components of loneliness and depressive symptoms. However, no evidence was found for changes in loneliness predicting changes in depressive symptoms, or vice versa (Griffin et al. 2022). Joshanloo (2022) separated between-person and within-person effects and investigated their influence on depression and loneliness over 3 and 6 years in a sample of German adults over 40. They found a reciprocal association between these variables at the 3-year lag but not at the 6-year lag, suggesting only short-term effects (Joshanloo 2022). Conversely, a study by Mayerl et al. (2023) investigated how loneliness and depressive symptoms in older adults unfolded together over 15 years. They found that both loneliness and depressive symptoms increased together at the same time within individuals, but found no evidence of short-term causal effects between them (Mayerl et al. 2023).

A working paper by Casabianca and Kovacic (2022) explicitly set out to identify causal effects of loneliness on a set of health outcomes, including depressive symptoms, among second-generation older immigrant adults. A specific maternal cultural background trait was used as an instrumental variable (IV) for loneliness to control for endogeneity. They found that loneliness was linked to an increased likelihood of depression, functional decline, and high body mass index, suggesting that there was a causal effect of loneliness on later health problems (Casabianca and Kovacic 2022). Additionally, a recent study by Sbarra et al. (2023) also set out to identify causal effects. They used several Mendelian Randomization (MR) analyses, using multiple independent genetic variants associated with loneliness as instrumental variables, to investigate the association between loneliness and major depression (MD) and found evidence of bidirectional causal associations between the two mental health concerns (Sbarra et al. 2023).

The explicit strategy for identifying causal effects sets the latter studies apart from most studies on loneliness and health in old age. Few other studies have devised strategies to explicitly and systematically account for all sources of potential confounding, limiting the potential to interpret the findings in causal terms (Hernán and Robins 2020). Examples of potential factors that could confound the observed associations between loneliness and depressive symptoms are social anxiety, personality traits (e.g., neuroticism and low extraversion), bereavement, poor physical health, and social isolation, as these all have been associated with an increased risk for both loneliness and depression (Levin and Stokes 1986; Cacioppo et al. 2006; Hawkley and Cacioppo 2010; Coyle and Dugan 2012; Lewis et al. 2014; Taylor et al. 2018).

To address the threats of causality, this study uses the counterfactual theory of causal inference to guide its methodology. This study will additionally employ an endogenous treatment-effects (ETE) model in its analysis, with which we will model the longitudinal loneliness-depressive symptoms association, while simultaneously accounting for selection into loneliness by including a model of the selection in the analysis (StataCorp 2023a). In addition, this ETE model allows for comparison of potential unexplained variance of the exposure model and the outcome model and thus enables the model to account for unmeasured confounding (StataCorp 2023b).

### Aims of the study

The aim of this study is to test for evidence of a causal effect of loneliness on depressive symptoms among European adults of 65 years and older. The research questions that this study is aiming to answer are as follows: (1) Is there a longitudinal association between loneliness and depressive symptoms among older adults in Europe during the period 2015 to 2017? (2) Does the association remain when adjusting for measured and unmeasured confounding?

## Method

### Data material

The study is based on analyses of secondary data from the Survey of Health, Ageing and Retirement in Europe (SHARE). This is a multi-disciplinary and cross-national panel study that has collected data of more than 150,000 individuals from over 28 European countries and Israel since 2004 (Börsch-Supan et al. 2013). It includes a wide range of variables covering various domains, such as health, family and social networks, economic status, and life events. To ensure comparability of data across countries, SHARE devotes particular attention to harmonizing the collected data. By harmonizing the data, SHARE provides a rich resource for cross-national research on aging and related issues (Sirven and Debrand 2008; Börsch-Supan et al. 2013).

**Fig. 1:**
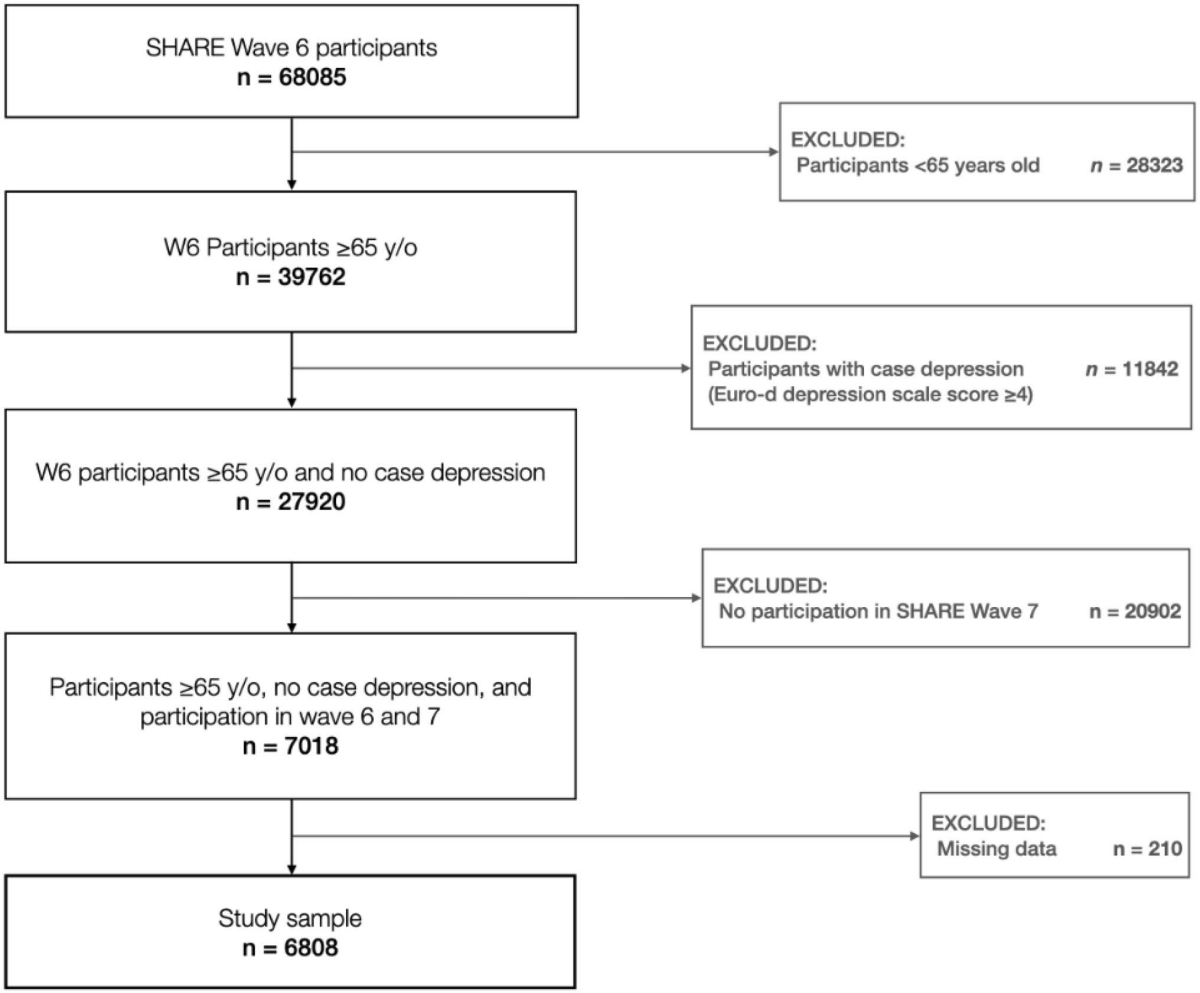
Flowchart describing the sample selection procedure

This study used data from SHARE Wave 6 and Wave 7, conducted in 2015 and 2017, respectively. The study sample was restricted to individuals aged 65 and older who were not depressed at baseline and participated in the main SHARE interview in both Wave 6 and Wave 7. Out of 68,085 observations at baseline, 61,277 were omitted because of the inclusion criteria, which resulted in an analytical sample of 6808 individuals for this study. See Fig. 1 for a description of the sample selection procedure.

### Depressive symptoms

The outcome variable, depressive symptoms, was measured by the Euro-D depression scale. This scale was developed to assess depression in later life in Europe and includes items such as depression, pessimism, irritability, sleep, and enjoyment (Prince et al. 1999). SHARE operationalizes depression with the generated variables EURO-D (eurod) and EURO-D caseness (eurodcat) which facilitate cross-national analysis and aim for a more accurate understanding of the phenomena being studied (SHARE-ERIC 2022). The eurod variable is a composite index measure of 12 questions that aim to capture the essence of depression symptoms (Mehrbrodt et al. 2021). These questions range from the direct “*In the last month, have you been sad or depressed?*” question, to more indirect questions like “*What has your appetite been like?*.” The maximum score is 12, indicating ‘very depressed,’ while the minimum score, 0, indicates ‘not depressed.’ The eurodcat variable was created to determine (non-clinical) depression. A score of 4 or more indicates case of depression, and a score below 4 indicates no depression. This method was based on the Prince et al. (1999) study, which suggests that a score of 3 or higher on the scale is suggestive of depression, while a score of 4 or higher indicates probable depression. As a result, the binary eurodcat variable was generated (1 = case depression, 0 = not depressed) (Mehrbrodt et al. 2021). Data from Wave 7 were utilized as the outcome measure for depressive symptoms. We tested the internal consistency and validity of the eurod variable in our sample by estimating the Cronbach’s alpha and a confirmatory factor analysis. The Cronbach’s alpha for the items was 0.63, which is within the range that has previously been observed in SHARE (Castro-Costa et al. 2008). Overall, the goodness-of-fit indices from the confirmatory factor analysis suggest that the Euro-D scale reasonably captures population-level patterns in depressive symptoms, despite limited inter-correlations among the included items (RMSEA: 0.055; SRMR: 0.042; CFI: 0.81; TLI: 0.76; CD: 0.657).

The depression caseness variable was used in Wave 6 to exclude participants that were depressed at baseline.

### Loneliness

In this study, we chose to incorporate both direct and indirect measures of loneliness. *Indirect* loneliness was measured using the Three-Item UCLA Loneliness Scale (UCLA-3), a widely recognized measure developed by Hughes and colleagues in 2004. This three-item scale measures indirect loneliness and thus excludes words like ‘lonely’ or ‘loneliness’ and instead focuses on respondent’s feeling isolated from others, left out, or a lack of companionship (Hughes et al. 2004). Experiences were rated via a three-point Likert scale with: ‘often,’ ‘some of the time,’ or ‘hardly ever or never,’ resulting in a score of 3 to 9 with a higher score meaning greater loneliness (Mehrbrodt et al. 2021). We tested the internal consistency and validity of the UCLA-3 index in our sample by estimating the Cronbach’s alpha and a confirmatory factor analysis. The Cronbach’s alpha for the items was 0.69. The fit indices from the confirmatory factor analysis are not useful as there were zero degrees of freedom, but the coefficient of determination (CD) showed that 73.5% of the variance in the items can be explained by the model. *Direct* loneliness was measured by respondent’s self-assessment of loneliness via the question “*How much of the time do you feel ****lonely***,” also answered on a three-point Likert scale with ‘often,’ ‘some of the time,’ or ‘hardly ever or never’ (Mehrbrodt et al. 2021). Including both these measures is hypothesized to capture a more accurate representation of the respondent’s experience of loneliness. As the analysis we use in this study includes a model of the selection into “treatment” (i.e., loneliness), we needed to dichotomize the loneliness measure. A clear cutoff point for an individual being ‘lonely’ or ‘not lonely’ has yet to be established and most studies merely dichotomize direct loneliness measures (Mund et al. 2020). Thus, specifically for this study, loneliness was defined as having multiple questions answered as ‘some of the time’ or at least one question answered as ‘often,’ resulting in a cutoff score of 5 or higher on the loneliness scale or a score of 1 (often) or 2 (some of the time) on the direct loneliness question. The data on loneliness were collected at baseline.

### Covariates

In order to account for potential confounding effects in the association between loneliness and depressive symptoms in old age, variables related to sociodemographic, health, functional and psychosocial factors were included in analyses. Data of these variables were collected at baseline and measured as follows. **Sociodemographic variables** included *age* (range = 65–99, in years), *sex* (0 = male, 1 = female), *partner loss* (0 = no, 1 = yes), *household size* (n individuals living in respondent's home), *country* (country where the SHARE interview took place), *foreign born* (0 = no, 1 = yes), participation in *social activities within the last year* (0 = no, 1 = yes). **Health and functional variables** included *instrumental activities of daily living*, *IADL*, an index used to assess limitations with nine instrumental everyday self-care activities like making a telephone call or taking medications (range = 0–9, A higher score indicates more difficulties and lower mobility), *global self-rated health* (0 = good, 1 = less than good), *multimorbidity* (0 = one or no chronic disease, 1 = 2 or more chronic diseases), *global activity limitation index*, GALI, measures disability or long-standing activity limitations due to general health problems (0 = not limited, 1 = limited), *physical inactivity* (0 = no, 1 = yes), *hearing status* (self-assessment of hearing, 0 = good to excellent hearing, 1 = less than good hearing), *vision status* (self-assessment of vision, 0 = good to excellent vision, 1 = less than good vision), *cognitive functioning* ranged from 1 to 5 with a higher score meaning less good memory, troubled by *pain* (0 = no, 1 = yes). **Socioeconomic variables** include *education* (1 = primary education, 2 = secondary education, and 3 = tertiary education, measured using the ISCED-97 coding), *self-reported financial distress* (0 = no, 1 = yes). Finally, to address extreme outliers, income was top coded for the top 1% of incomes. Additionally, for an accurate representation of financial resources available per person, the total household income was adjusted for household size using the square root scale method (OECD 2023).

### Ethical considerations

The research in this study has been approved by the Swedish Ethical Review Authority (dnr. 2020-00964).

### Data analyses

**Fig. 2:**
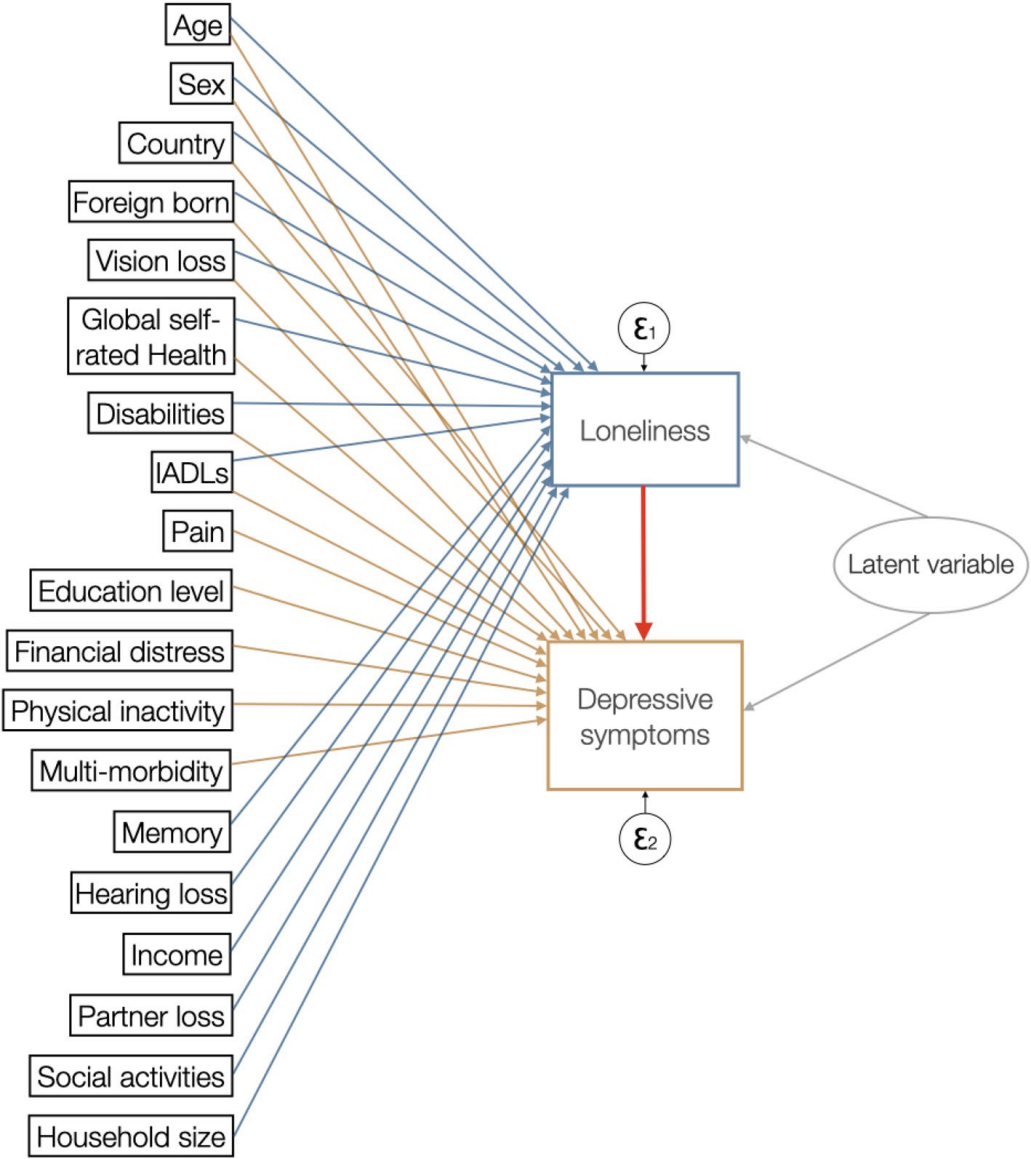
Visualization of the endogenous treatment-effects model to test for evidence of a causal effect of old age loneliness on depressive symptoms

Data management was done using R-STUDIO version 2023.03.0 + 386, statistical analyses were performed in Stata/SE 17.0 and Fig. 2 is made in keynote. Uni- and bivariate descriptive statistics were generated and are presented in Tables 1 and 2. The longitudinal multivariate analyses were performed in three steps: (1) simple linear regression, to investigate if there was an association between loneliness and depressive symptoms among older adults in Europe during the period 2015 to 2017; (2) multivariable linear regression to examine if this relation persists when controlling for basic demographic compositional differences (age, sex, and country); (3) the ETE-model, which estimates the causal effect of later life loneliness on depressive symptoms while accounting for potential unmeasured confounding factors and endogeneity issues. The ETE is modeled as a generalized structural equation model, where selection into “treatment” (i.e., loneliness), and the effect of loneliness on subsequent depressive symptoms is modeled simultaneously. To examine and account for unmeasured confounding, the model adjusts for the covariance of the residuals from both the selection model and the outcome model (Drukker 2016). By post-estimations, it is in turn possible to test whether there were unobserved variables that statistically significantly confounded the effect of loneliness on depressive symptoms (StataCorp 2023a).

**Table 1:** Weighted descriptive statistics of our sample. Covariates are described at baseline and specifically for loneliness and depressive symptoms. n = 6808

Continuous variables	Baseline (Wave 6)	Loneliness (Wave 6)	Case depression (Wave 7)
	Mean (SD)		
Age	74.01 (6.78)	75.63 (7.14)	76.15 (7.38)
Income	18,602.67 (14,281.52)	15,227.63 (12,383.65)	15,711.18 (12,451.94)
Limitations with IADL	0.28 (0.95)	0.42 (1.13)	0.54 (1.31)
Memory test score	3.09 (0.88)	3.17 (0.92)	3.30 (0.92)
Household size	1.93 (0.89)	1.66 (0.88)	1.98 (0.97)
Euro-d depression scale score	1.37 (1.09)	1.72 (1.05)	5.23 (1.47)

Categorical variables	% (n)	% lonely per category (n)	% depressed per category (n)
Sex			
Male	49.21 (3350)	23.04 (773)	15.49 (519)
Female	50.79 (3458)	32.36 (1119)	23.51 (813)
Partner loss			
Yes	20.72 (1411)	49.18 (694)	25.66 (362)
No	79.28 (5397)	22.20 (1198)	17.99 (971)
Highest level of education			
Primary	34.06 (2319)	35.88 (832)	27.90 (647)
Secondary	43.64 (2971)	24.54 (729)	16.46 (489)
Tertiary	22.30 (1518)	21.74 (330)	12.91 (196)
Financial distress			
Yes	28.62 (1949)	39.40 (768)	25.35 (494)
No	71.38 (4859)	23.11 (1123)	17.27 (839)
Born in country of interview			
Yes	94.04 (6402)	27.52 (1762)	19.51 (1249)
No	5.96 (406)	24.25 (130)	20.69 (84)
Multimorbidity			
Yes	54.25 (3693)	29.84 (1102)	23.67 (874)
No	45.75 (3115)	25.33 (789)	14.70 (458)
Long-standing activity limitations (GALI)			
Yes	40.63 (2766)	33.19 (918)	25.16 (696)
No	59.37 (4042)	24.07 (973)	15.76 (637)
Social activities within the last year			
Yes	39.32 (2677)	22.30 (597)	13.75 (368)
No	60.68 (4131)	31.32 (1294)	23.36 (965)
Global self-rated health			
Very good/excellent	20.88 (1422)	17.93 (255)	8.37 (119)
Less than very good	79.12 (5386)	30.39 (1637)	22.54 (1214)
Hearing status			
Less than good	21.64 (1473)	32.11 (473)	24.58 (362)
Good or excellent	78.36 (5335)	26.58 (1418)	18.18 (970)
Vision status			
Less than good	17.53 (1193)	38.56 (460)	30.60 (365)
Good or excellent	82.47 (5615)	25.49 (1431)	17.24 (968)
Bothered with pain			
Yes	42.86 (2918)	33.82 (987)	26.29 (767)
No	57.14 (3890)	23.24 (904)	14.55 (566)
Physical inactivity			
Yes	9.98 (701)	37.38 (262)	36.66 (257)
No	90.02 (6107)	26.67 (1629)	17.62 (1076)
Lonely			
Yes	27.78 (1891)	100 (1873)	28.34 (536)
No	72.22 (4917)	0 (0)	16.19 (796)

**Table 2:** Bivariate (Model 1) and multivariate regression (Model 2) of depressive symptoms on loneliness with coefficients, significance levels (p), 95% confidence intervals, and R-squared for explained variance^a^

Variable	Coefficient	Robust std. err	*p*	95% CI	Coefficient	Robust std. err	*p*	95% CI
	Model 1				Model 2			
Loneliness	0.750	0.084	0.001	[0.584, 0.915]	0.563	0.086	0.000	[0.394, 0.731]
Intercept	1.855	0.039	0.000	[1.779, 1.930]	− 2.880	0.431	0.000	[3.720, 2.030]
ther statistics:								
*R*^2^	0.0295				0.0939			

^a^ Model 2 was adjusted for: age, sex and country

One caveat with this model is that it uses an OLS estimation, which could be problematic given that the outcome (depressive symptoms) is a limited dependent variable (bounded and skewed). Yet, there is evidence that OLS regressions perform well even when distributional assumptions about the outcome variable are violated, when the goal is to estimate an average effect (Angrist and Pischke 2009). In addition, previous studies have shown similar patterns from models using OLS and GLM estimations with eurod as outcome (cf. Sjöberg 2023). We used robust standard errors to account for heteroscedasticity concerns.

In addition, all models were adjusted for the clustering of observations within countries. Lastly, calibrated cross-sectional weights for Wave 7, provided from the SHARE team, were used to adjust for sample structure in the survey data. This was done to ensure that the sample was representative of the target population and to improve the accuracy of estimates and inferences. Based on the study design and choice of weights, the target population for inference was the population from the included countries, aged 67 year and older in 2017, with no case depression in 2015.

### Missing data

To address item non-response and errors in the data, SHARE provides imputed variables. These imputed variables are carefully created by SHARE and more information on the exact methods can be found in their release guide provided with each data wave release (SHARE-ERIC 2022). In our sample, the following variables (number of imputed cases in parenthesis) were affected by SHARE imputations: income (2387), education (239), eyesight (2), hearing (1), physical inactivity (1), memory (891), financial distress (65), social activities within the last year (1), and depressive symptoms (152). This study used these imputed variables to achieve a more complete dataset, more efficient analyses, and increase overall statistical power. We generated five imputed datasets and pooled estimates using Rubin’s Rules, which accounted for both within- and between-imputation variability. The pooled coefficients, standard errors, confidence intervals, and p values are reported.

## Results

### Sample

Table 1 shows the weighted descriptive statistics of our sample. The 6808 participants were an average of 74.01 years old (SD = 6.78) and lived in a household with an average of 1.93 (SD = 0.89) people; the sample does not include individuals living in a nursing home. There was a slight majority of female participants (50.79%). Most had secondary levels or tertiary levels of education (65.94%), were born in the interview country (94.04%), and did not participate in any social activities within the last year (60.68%). The average top-coded equalized income was 18,602.67 Euros (SD = 14,281.52). More than half of the participants suffered from two or more chronic conditions (multimorbidity; 54.25%), but less than half (40.63%) had long-standing activity limitations or disabilities (GALI). The average score of a memory test was 3.09 (SD = 0.88) on a scale ranging from 1 to 5, with a higher score indicating less good memory. Health was rated less than very good by most participants (79.12%); however, hearing was considered good or excellent by most (78.36%) as well as their vision (82.47%), and just over 57% of the participants were not bothered with pain. The average score on limitations with IADLs was 0.28 (SD = 0.95), and a large majority of the individuals in the sample were physically active (90.02%). Finally, at baseline (Wave 6) more than a quarter of individuals reported feeling lonely at least some of the time (27.78%) and the score on the depression scale was on average 1.37 (SD = 1.09). At wave 7—outcome measure of depression—in for those considered depressed, the mean score was 5.23 (SD = 1.47). A country specific table for frequencies, loneliness and depressive symptoms was also made and can be found in appendix (Table A1).

### The association between old age loneliness and depressive symptoms

Table 2 shows the weighted bi- and multivariate regressions of depressive symptoms on loneliness (age, sex, and country). The regression models investigate the longitudinal association between loneliness and depressive symptoms among older adults in Europe during the period 2015 to 2017 and show that loneliness is positively and statistically significantly related to subsequent depressive symptoms in our sample. Model 1 only explains 2.95% of the variance in depressive symptoms (*R*^2^ = 0.0295); for Model 2, the explained variance is 9.39% (*R*^2^ = 0.0939). The positive relationship between loneliness and subsequent depressive symptoms persists even after controlling for age, sex, and country, and as the coefficient estimate in Model 2 is lower than in Model 1, some of this association could be attributable to the composition of these variables. Overall, the results indicate that older adults who were lonely at baseline were at an increased risk of subsequent depressive symptoms in comparison with older adults who were not lonely. In the bivariate model, being lonely at baseline was associated with 0.75 more depressive symptoms at follow-up, compared to those who were not lonely at baseline (Model 1: *β* = 0.750, SE = 0.084, *p* < 0.001). After adjusting for age, sex, and country, the excess symptomology observed among the lonely was reduced to 0.56 symptoms, on average (Model 2: *β* = 0.563, SE = 0.086, *p* < 0.001).

### Testing the predictive power of the associated factors

To assess the predictive power of the associated factors, separate models for old age loneliness and depressive symptoms were made and their estimates are shown in Table A5 of appendix. Besides being predictive, these models illustrate their functioning in the ETE analysis. For loneliness, the overall model is statistically significant (*p* < 0.05) and explains about 14.4% of the variance. For depressive symptoms, the overall model is also statistically significant (*p* < 0.01) and explains about 13.3% of the variance. From this, we conclude that the included factors do predict loneliness or depressive symptoms, albeit only partially.

### The endogenous treatment-effects estimates

Figure 2 visualizes how the ETE-model works, and Table 3 shows its analysis results. The average treatment effect (ATE) of old age loneliness on depressive symptoms, which is the difference in the mean outcome between those who were lonely at baseline and those who were not, is 0.228. This means that loneliness is associated with an increase in depressive symptomatology by 0.228 units, on average; however, this finding is not statistically significant (*p* = 0.280).

**Table 3:** ETE-model analysis estimates for investigating the ‘treatment-effect’ of old age loneliness on depressive symptoms with post-estimation of rho to test for endogeneity^a^

	Coefficient	Robust standard error	*P* > *z*	Confidence interval (95%)	
				Low	High
Loneliness (yes)	0.228	0.183	0.280	− 0.131	0.586
Rho	0.0705	0.053	0.251	− 0.033	0.174

^a^Adjusted for: age, sex, country, foreign born, vision, global self-rated health, longstanding disabilities, IADL, pain, education, financial distress, physical inactivity, multimorbidity, memory, hearing, income, social activities, partner loss, household size

A post-estimation of rho was done to test for endogeneity. It tests whether unobserved factors, represented by the latent variable, are correlated with loneliness and depressive symptoms. The null hypothesis in this post-estimation is that loneliness and depressive symptom unobservables are uncorrelated. The estimation shows that the rho-coefficient is 0.071, and the *p* value is 0.251. Thus, there is not enough evidence to conclude a correlation between the errors in the treatment and outcome equations. Therefore, the null hypothesis cannot be rejected and there is no reliable evidence of unmeasured confounding in the specified model.

## Discussion

The results from this study show that there was a substantial and significant positive longitudinal association between loneliness and subsequent depressive symptoms in our sample. This association remained statistically significant after adjusting for age, sex, and country of interview. However, after adjusting for potential measured and unmeasured confounding, the effect size was small and statistically non-significant, yielding little evidence for loneliness being a substantial cause of incident depressive symptoms in old age. Finally, after testing for endogeneity, little evidence was found for unmeasured confounding affecting the relationship, suggesting that the confounding was sufficiently accounted for by the manifest variables included in our model. Overall, the findings confirm that there is a substantial association between loneliness during old age and later depressive symptoms; however, it does not seem to be driven by a causal effect of loneliness on the risk of depressive symptoms. Rather, loneliness seems to primarily work as an indicator of other living conditions that are associated with an increased vulnerability to depressive symptoms.

As all studies, this study has some limitations, and the findings should be interpreted with caution. For example, we merged data from various European countries, which may have obscured the relationship between old age loneliness and later depressive symptoms within specific sociocultural contexts. This approach could have homogenized the findings and hidden potential variations in the association between the two mental health concerns between different countries. In addition, our study focused on a specific two-year period (2015–2017) and examined older (65+) individuals who were not depressed at baseline. These specifics might limit the generalizability of our findings to the broader population. Moreover, the limited timeframe may not be sufficient to fully capture the longitudinal relationship between loneliness and depressive symptoms in older adults. On the other hand, attrition during follow-up may have influenced the results, particularly if it was selective with respect to factors relevant to this study. To assess this, we compared the characteristics of the full sample of eligible participants with available data at wave 6 to those who participated in both waves 6 and 7 across a series of variables central to this study. We found little evidence of systematic differences in composition. However, it remains possible that attrition was selective for unmeasured factors that are nonetheless important for the results.

Certain aspects of the study design may also have affected the estimated associations. These include the limited internal consistency of both the loneliness and depressive symptom scales, the dichotomization of the loneliness measure, and the use of OLS regression for a skewed and bounded outcome variable. Together, these factors could contribute to an artefactual attenuation of the true associations.

Although differences in measures and study designs complicate direct comparisons with other studies, our findings still demonstrate statistically and clinically significant differences in depressive symptoms between the groups. This holds true in both bivariate models and models adjusted for age, sex, and country, despite the noted limitations. Finally, this study did not rely on a clinical diagnosis of depression, nor does there exist a standardized common set of valid measures for loneliness. The EURO-D Scale was used as measurement of depressive symptoms. It is based on participants’ own assessment of their symptoms in relation to the survey questions. The same is true for the three-item UCLA Loneliness Scale complemented by a direct, single question, loneliness measure to create a cutoff point for dichotomizing loneliness. This approach is based on sound reasoning and face-validity, but the specific criteria used to define loneliness and the cutoff scores for identifying loneliness can of course influence the results. The crudeness of the measures may have oversimplified the complex nature of the two mental health concerns.

The primary strength of this study is that, to our knowledge, it is the first in its kind to model the longitudinal loneliness-depressive symptoms association in old age while accounting for unmeasured confounding through the comparison of potential unexplained variance from the exposure- and outcome models. Therefore, the study was able to investigate the causal effect of loneliness on depressive symptoms in older adults. With this, our study answers to a call to investigate to which extent the association between old age loneliness and, in our case, depressive symptoms, reflects causal effects of loneliness rather than confounding by unmeasured variables (Ong et al. 2016). Moreover, this study measured the exposure variable loneliness and covariates at baseline (among older adults without depressive symptoms), and the outcome variable depressive symptoms at later time, and thus accounted for the temporal order of exposure and outcome. Hence, the study was designed to mimic an experimental study design despite being of observational nature and to rule out as much reversed causation from pre-existing depressive symptoms as possible.

The findings in our study are consistent with those of previous studies concerning the substantial positive association between old age loneliness and depressive symptomatology (Heikkinen and Kauppinen 2004; Cacioppo et al. 2006; Powell et al. 2021; Luo 2023) and suggest that lonely older adults are at an increased risk of developing depressive symptoms. Also congruent with prior studies is that loneliness and depressive symptoms vary across subgroups of the sample, with higher rates in women for both mental health concerns (Maier et al. 2021; Dahlberg et al. 2022). However, we found limited evidence for a causal effect of old age loneliness on depressive symptoms in our sample. This stands in contrast with the recent studies of Casabianca and Kovacic (2022) and Sbarra et al. (2023) who both did find evidence for a causal effect of loneliness on depressive symptoms. While they explicitly devised strategies to find causal effects and systematically accounted for endogeneity, their methods differed from ours as both studies made use of instrumental variable strategies. While Sbarra et al. (2023) attempted to account for pleiotropy by using multiple independent genetic variants associated with loneliness as IVs, their findings could still be influenced by genetic confounding. As existing research underscores the trait-like nature of both loneliness and depression (Griffin et al. 2022; Mayerl et al. 2023), a potential genetic overlap between the two mental health concerns is plausible. That is, the genetic instruments associated with loneliness might also have some direct influence on depression, which raises questions about the extent to which the true causal relationship can be isolated using this particular MR approach. The IV in the Casabianca and Kovacic (2022) study (a specific maternal cultural background trait) potentially only captures one specific dimension of loneliness. Therefore, their results may not be generalizable to all-cause loneliness. However, more in line with our findings are those of Wootton et al. (2021) who utilized MR to assess bidirectional causal effects between loneliness, smoking, and alcohol use. They did not find strong evidence for loneliness causing the studied negative health behaviors, while also employing a causal inference approach (Wootton et al. 2021).

Our findings show that lonely older adults are at an increased risk of developing depressive symptoms, yet the association does not seem to primarily be an effect of loneliness in itself. These findings suggest that loneliness is primarily an indicator of an increased susceptibility to depressive symptoms in older individuals, for example, due to vulnerable living conditions or psychological predispositions. Thus, as our study challenges the notion that old age loneliness is a causal factor of later depressive symptoms, future research should use different methods and data to test the robustness of our findings. By having different approaches, unrelated sources of bias, and triangulating the results, confidence in the results would be strengthened (Lawlor et al. 2016). Additionally, future theoretical and empirical work should focus on identifying the underlying mechanisms that contribute to the increased susceptibility to both loneliness and depressive symptoms among certain older adults. These mechanisms could include life events such as bereavement or the onset of disabilities, as well as psychological vulnerabilities such as social anxiety and neuroticism.

To conclude, to our knowledge, this study is the first to use an endogenous treatment-effects model to examine the causal relationship between loneliness and depressive symptoms in the 65+  population of Europe. Despite finding a substantial positive association between loneliness in 2015 and depressive symptoms in 2017, little evidence for a causal effect of loneliness on subsequent depressive symptoms was found. The findings suggest that lonely older adults are at an increased risk of developing depressive symptoms. Yet, this does not seem to primarily be a direct effect of the loneliness itself, but rather of other precarious living conditions associated with feelings of loneliness.

## Supplementary Information

Below is the link to the electronic supplementary material.

Supplementary file 1 (DOCX 36 KB)

## Data Availability

All analyses are conducted using data publicly available via: https://share-eric.eu/
