# Supplementary material for: The effect of loneliness on depressive symptoms in the 65+ European population: a longitudinal observational study using SHARE data
Source: Eur J Ageing. 2025 Mar 13;22(1):9. doi: 10.1007/s10433-025-00846-0 (PMC11904008; doi:10.1007/s10433-025-00846-0)
Supplement: Supplementary file 1 — (DOCX 36 KB) [file 10433_2025_846_MOESM1_ESM.docx]

# Appendix

## List of tables

Table A1. Country specific frequency table

Table A2. Predictors of loneliness in old age

Table A3. Predictors of depression in old age

Table A4. Internal consistency and validity of the Loneliness Scale and Euro-D depression scale

Table A5. Predictive models of loneliness and depression in old age

Table A6. Weighted descriptive statistics and attrition analyses

| **Table A1.** Country specific frequency table, including frequencies and percentages specified for loneliness and case depression per country | | | | | | |
| --- | --- | --- | --- | --- | --- | --- |
|  | Total Sample | | Lonely  (Wave 6) | | Case depression  (Wave 7) | |
| **Country** | **Frequency** | **Percent (%)** | **Frequency** | **Percent (%)** | **Frequency** | **Percent (%)** |
| Austria | 77 | 1.13 | 13 | 16.88 | 11 | 14.29 |
| Germany | 1454 | 21.36 | 301 | 20.70 | 204 | 14.03 |
| Sweden | 239 | 3.38 | 75 | 31.38 | 30 | 12.55 |
| Spain | 1079 | 15.86 | 260 | 24.10 | 255 | 23.63 |
| Italy | 1264 | 18.57 | 521 | 41.22 | 309 | 24.44 |
| France | 1154 | 16.96 | 243 | 21.06 | 228 | 19.76 |
| Denmark | 175 | 2.57 | 26 | 14.86 | 20 | 11.43 |
| Greece | 372 | 5.46 | 196 | 52.69 | 68 | 18.28 |
| Switzerland | 171 | 2.51 | 28 | 16.37 | 21 | 12.28 |
| Belgium | 253 | 3.72 | 61 | 24.11 | 44 | 17.39 |
| Czech Republic | 179 | 2.63 | 51 | 28.49 | 27 | 15.08 |
| Poland | 399 | 5.87 | 116 | 29.07 | 115 | 28.82 |
| Total | 6808 | 100 | 1891 |  | 1332 |  |

| **Table A2.** By reviewing the existing literature, we identified factors associated with loneliness in older adults for final use in our analyses. They are represented in the table below. The table shows the name of the predictor, the direction of association with depression, and its source reference. | | |
| --- | --- | --- |
| **Predictor** | **Association with loneliness** | **Source** |
| Age | **+** | Dahlberg et al. 2022 |
| Income | **-** |  |
| Limitations with IADL | **+** |  |
| Less than good memory | **+** |  |
| Household size | **-** |  |
| Sex (ref. man) | **+** |  |
| Partner loss | **+** |  |
| Long-standing activity limitations (GALI) | **+** |  |
| Social activities | **-** |  |
| Less than very good global self-rated health | **+** |  |
| Less than good hearing | **+** | Wang et al. 2022 |
| Less than good vision | **+** |  |
| Depression | **+** | J. T. Cacioppo et al. 2006, 2010; Dahlberg et al. 2022; Wister et al. 2022 |
| Country |  | Heu et al. 2021; Lykes & Kemmelmeier 2014 |

| **Table A3.** By reviewing the existing literature, we identified factors associated with depression in older adults for final use in our analyses. They are represented in the table below. The table shows the name of the predictor, the direction of association with depression, and its source reference. | | |
| --- | --- | --- |
| **Predictor Name** | **Association with depression** | **Source** |
| Age | **+** | Wister et al. 2022 |
| Foreign born | **+** |  |
| Limitations with IADL | **+** |  |
| Financial distress | **+** |  |
| Less than very good global self-rated health | **+** |  |
| Physical activity | **-** | Maier et al. 2021 |
| Long-standing activity limitations (GALI) | **+** | deducted from Maier et al. 2021 |
| Sex (ref. man) | **+** | Maier et al. 2021; Wister et al. 2022 |
| Education level (ref. primary education) | **-** |  |
| Bothered with pain | **+** | Heikkinen & Kauppinen 2004; Maier et al. 2021; Wister et al. 2022 |
| Multimorbidity | **+** |  |
| Less than good vision | **+** |  |
| Country |  | Heu et al. 2021; Lykes & Kemmelmeier 2014 |

| **Table A4.** Internal consistency and validity of the Loneliness Scale and Euro-D depression scale | | | |
| --- | --- | --- | --- |
|  | **Loneliness Scale** |  | **Depression Scale** |
| **Cronbach’s Alpha** |  | | |
| Average interitem correlation | 0.425 |  | 0.125 |
| Number of items in the scale | 3 |  | 12 |
| Scale reliability coefficient | 0.689 |  | 0.631 |
| **SEM Goodness-of-Fit statistics** |  |  |  |
| Chi-Square Test | 0.022 (0.124) |  | 1148.71 (p<0.001) |
| Root Mean Squared Error of  Approximation (RMSEA) | 0.000 |  | 0.055 |
| CFI | 1.000 |  | 0.807 |
| TLI | 1.000 |  | 0.764 |
| Standardized Root Mean Squared Residual (SRMR) | 0.000 |  | 0.042 |
| Coefficient of Determination (CD) | 0.735 |  | 0.657 |
|  | | | |

| **Table A5.** Weighted predictive models for loneliness and depression with associated predictor variables based on the literature review. A linear regression model for depressive symptoms (Model 1) and logistic regression model for loneliness (Model 2) with coefficients, standard errors (SE), and significance levels (*) are shown in the table below. | | | |
| --- | --- | --- | --- |
|  | Model 1 |  | Model 2 |
| **Variable** | **Coefficient (SE)** |  | **Coefficient (SE)** |
| Age | 0.033 (0.006)*** |  | 0.007 (.008) |
| Sex | 0.394 (0.069)*** |  | 0.095 (.095) |
| Country |  |  |  |
| Germany | 0.143 (0.144) |  | 0.252 (0.248) |
| Sweden | 0.062 (0.133) |  | 1.080 (0.224)*** |
| Spain | 0.556 (0.156)*** |  | 0.676 (0.244)** |
| Italy | 0.393 (0.161)** |  | 1.510 (0.229)*** |
| France | 0.289 (0.139)* |  | 0.476 (0.236)* |
| Denmark | 0.098 (0.130) |  | 0.004 (0.239) |
| Greece | 0.059 (0.153) |  | 2.130 (0.224)*** |
| Switzerland | 0.0762 (0.137) |  | 0.202 (0.254) |
| Belgium | 0.240 (0.131) |  | 0.756 (0.222)*** |
| Czech Republic | 0.044 (0.160) |  | 0.772 (0.245)** |
| Poland | 0.617 (0.175)*** |  | 0.857 (0.258)*** |
| Foreign born | 0.235 (0.161) |  | 0.468 (0.205)* |
| Vision status | -0.336 (0.097)*** |  | -0.377 (0.116)*** |
| Global self-rated health | 0.375 (0.076)*** |  | 0.308 (0.125)* |
| GALI | 0.241 (0.079)** |  | 0.332 (0.099)*** |
| IADL limitations | 0.057 (0.050) |  | 0.055 (0.060) |
| Pain | 0.313 (0.076)*** |  |  |
| Highest attained education |  |  |  |
| secondary education | -0.232 (0.089)** |  |  |
| tertiary education | -0.279 (0.095)** |  |  |
| Financial distress | 0.129 (0.085) |  |  |
| Physical inactivity | 0.422 (0.149)** |  |  |
| Multimorbidity | 0.139 (0.071)* |  |  |
| Memory |  |  | 0.028 (0.055) |
| Hearing status |  |  | -0.208 (0.113)* |
| Income |  |  | -0.000 (0.000)** |
| Social activities in last year |  |  | -0.153 (0.103) |
| Partner loss |  |  | 0.864 (0.132)*** |
| Household size |  |  | -0.559 (0.079)*** |
| Intercept (full model) | -1.550 (0.484)** |  | -1.420 (0.689)* |
| Other statistics | R^2^= 0.1330 |  | Pseudo R^2^ = 0.1440 |

* p < .05; ** p < .01; *** p<.001
***^a^*** *The overall model for depression (model 1) is statistically significant (p<0.01) and explains about 13.30% of the variance in depressive symptoms. The overall model for loneliness (model 2) is also statistically significant (p<0.05) and explains about 14.40% of the variance in loneliness. Both models illustrate that the chosen factors do actually predict loneliness and depression albeit only partially.*
***^b^*** *Reference category country: Austria*
***^c^*** *Reference category education: primary education*

| **Table A6.** Weighted descriptive statistics and attrition analyses. Sample n=6808 and excluded data n=19640. | | | |
| --- | --- | --- | --- |
|  | Sample (Wave 6) | Excluded data (Wave 6) | Attrition analysis |
| **Continuous variables** | **Mean (SD)** | | **p-value** Welch t-test |
| Age | 73.96 (6.81) | 74.28 (7.06) | 0.996 |
| Income | 17954.63 (14173.98) | 18957.15 (14283.15) | 0.871 |
| Euro-d depression scale score | 1.39 (1.10) | 1.41 (1.09) | 0.984 |
| **Categorical variables** | **% (n)** | **% (n)** | **p-value** Pearson’s Chi²-test |
| Sex |  |  |  |
| Male | 49.78 (3389) | 50.36 (9890) | 0.412 |
| Female | 50.22 (3419) | 49.64 (9750) |  |
| Highest level of education |  |  |  |
| Primary | 34.61 (2357) | 29.90 (5872) |  |
| Secondary | 43.38 (2953) | 46.70 (9172) | 0.000 |
| Tertiary | 22.01 (1498) | 23.33 (4596) |  |
| Global self-rated health |  |  |  |
| Very good/excellent | 19.90 (1355) | 20.01 (3930) | 0.849 |
| Less than very good | 70.10 (5453) | 79.99 (15710) |  |
| Lonely |  |  |  |
| Yes | 27.51 (1873) | 27.20 (5343) | 0.624 |
| No | 72.30 (4935) | 72.80 (14297) |  |
| ***^a^*** *Wave 6 weights* ***^b^*** *p-values > 0.05 = no statistically significant difference between the two groups* | | | |
